# Assessment of Maternal Knowledge and Practices Regarding Acute Diarrheal Illnesses in Children in Saudi Arabia: A Tertiary Care Center Survey

**DOI:** 10.7759/cureus.33116

**Published:** 2022-12-29

**Authors:** Abdulaziz S Alrafiaah, Ahmed Albraikan, Azzam AlJaafari, Abdullah A Alabbad, Hamad Alfehaid, Sulaiman Alqueflie, Aamir Omair

**Affiliations:** 1 Department of Pediatrics, Majmaah University, Al Majma'ah, SAU; 2 Department of Pediatrics, King Abdullah Specialist Children Hospital, Riyadh, SAU; 3 Department of Medical Education, King Saud bin Abdulaziz University for Health Sciences, Riyadh, SAU

**Keywords:** gastroenteritis in children, saudi arabia, maternal knowledge, oral rehydration solution, infectious diarrhea

## Abstract

Background

Diarrheal illness remains one of the most common causes of death among children specifically those who live in developing countries. Appropriate mothers' knowledge and practice regarding acute diarrhea illness in children can considerably reduce diarrhea-related morbidity and mortality. So, the aim of this study was to evaluate mothers’ knowledge and practice regarding acute diarrheal illness in children.

Method

This cross-sectional study was conducted in a tertiary hospital in Riyadh, Saudi Arabia using a newly developed questionnaire that was distributed to King Abdullah Specialist Children. The calculated sample size was 375.

Results

A total of 375 mothers were included in this study and the majority of them (99%) were Saudis. More than half of the participants (61%) were university graduates. The majority (96.5%) chose previous experience as the main source of information about diarrhea while (40%) chose physician. Most of the mothers (69.6%) believed that teething is the leading cause of diarrheal illness in children. Regarding the treatment, fluid was recognized to be the major treatment for diarrhea as chosen by the mothers. Around (42.7%) of the respondents thought intravenous fluid is more effective than oral rehydration solution (ORS) in treating dehydration associated with diarrheal episodes. ORS was the main type of fluid (74.1%) which was selected by the mothers to be given during the diarrheal episode.

Conclusion

Mothers showed good knowledge and practice overall in regard to acute diarrheal illness in children. However, this study revealed some misconceptions among the caregivers which necessitates more educational sessions to be conducted in the community and during the hospital visit.

## Introduction

Diarrhea is a major health problem and is one of the main causes of death among children less than five years of age [1]. It is defined as the passage of three or more loose or watery stools per day [2]. The burden of diarrheal illnesses is more noticeable in children who are originally from developing countries, and it is estimated that 1.3 million deaths annually are attributed to diarrhea [3,4]. Acute gastroenteritis, which is the most common cause of acute diarrheal illness in children, is accountable for almost 6% of all admissions to hospitals in children under 15 years [5]. Most diarrheal illnesses are caused by viral infections, with rotavirus being the most common infective agent [6].

Since the introduction of oral rehydration solution (ORS), the incidence of morbidity, mortality, and hospital admissions related to diarrhea, decreased significantly [7,8]. Management of acute diarrheal illness at home is one of the essential primary steps to prevent possible consequences of diarrhea such as dehydration [9,10]. The knowledge and practice of the parents in regard to the management of diarrheal illnesses play a significant role in the reduction of diarrhea-related complications [11]. Multiple studies were conducted globally to examine these issues and showed poor results in regard to maternal practices and knowledge of diarrheal diseases in children [12,13].

Due to cultural differences and understanding of the issue, maternal knowledge and practices regarding acute diarrheal illness in children in Saudi Arabia were examined in this study based on a tertiary care setting.

## Materials and methods

This cross-sectional study was conducted in a tertiary hospital in Riyadh, Saudi Arabia. The self-administered questionnaire was developed by the authors and was distributed to the mothers who visited King Abdullah Specialist Children Hospital. Informed consent was taken from the participants before participating in the study. All obtained data were confidential and were used only for scientific purposes. Institutional review board (IRB) approval was obtained from King Abdullah International Medical Research Center (IRB registration number H-01-R-005). The questionnaire was distributed in the Arabic language to mothers who visited King Abdullah Specialist Children Hospital’s emergency department, inpatient wards, and outpatient clinics. It is one of the biggest tertiary referral centers for children in the Kingdom of Saudi Arabia and in the gulf area.

Inclusion criteria and exclusion criteria

Any Saudi or non-Saudi mothers that visited King Abdullah Specialist Children Hospital, who had children below five years of age with a previous history of acute diarrhea (< 14 days), and who speak Arabic was included in the study. Participants who did not meet all inclusion criteria for the study were excluded.

Sampling technique and sample size calculation

The calculation of the sample size was done using a Raosoft sample size online calculator (Raosoft, Inc., Seattle, WA​​). To calculate the sample size, we used a 50% response distribution, a 5% margin of error, and a 95% confidence interval. The calculated sample size was 375. This was convenience sampling the questionnaire was distributed to mothers who met the inclusion criteria of the study.

Data collection methods and instruments used

A new questionnaire was developed by the authors after a comprehensive review of the related literature. The questionnaire was initially reviewed by two independent experts in the field of pediatric medicine as the first stage of the validation process. This phase was conducted to ensure the simplicity, precision, and relevance of the questionnaire’s items. After the initial validation process by the expert, the final questionnaire was included in a pilot study to complete the final validation process. Those participants who participated in the pilot study for the questionnaire validation were not included in the final analysis of the study.

The final validated questionnaire was divided into three sections and is composed of 14 questions. It aimed to test the mother’s knowledge, and practices regarding acute diarrheal illnesses in children in Saudi Arabia. The first part of the questionnaire was composed of four questions that were related to the demographic characteristics of the mothers such as nationality, age, educational level of the mothers, and the number of children. The second section was composed of seven questions that assessed the knowledge of the mother regarding diarrheal illnesses in children. Finally, the last part included three questions that showed the practices of the mothers regarding the management of diarrheal illnesses.

Statistical analysis

Data analysis was performed using Statistical Product and Service Solutions (SPSS) (IBM SPSS Statistics for Windows, Version 22.0, Armonk, NY). Descriptive statistics were calculated to evaluate the baseline demographics and socioeconomic factors of the participants. The categorical variables were presented as frequencies and percentages.

## Results

The number of mothers who participated in this study was 375 mothers who answered all of the questions. The demographic characteristics of these mothers are presented in Table 1. The majority of these mothers (99%) were Saudis. Almost half of the mothers (45%) are aged between 30 and 39 years. More than half of the participants (61%) were university graduates and 36% of the included sample had one to two children.

**Table 1:** Demographic characteristics of the mothers (N=375)

	n	%
Nationality		
Saudi	370	99%
Non-Saudi	5	1%
Age		
18-29 years	99	26%
30-39 years	167	45%
40+ years	109	29%
Educational status		
Illiterate	6	2%
Primary	12	3%
Intermediate	30	8%
High School	82	22%
University	228	61%
Postgraduate	17	5%
Number of children		
1-2	136	36%
3-4	114	30%
5+	125	33%

Knowledge

**Figure 1:**
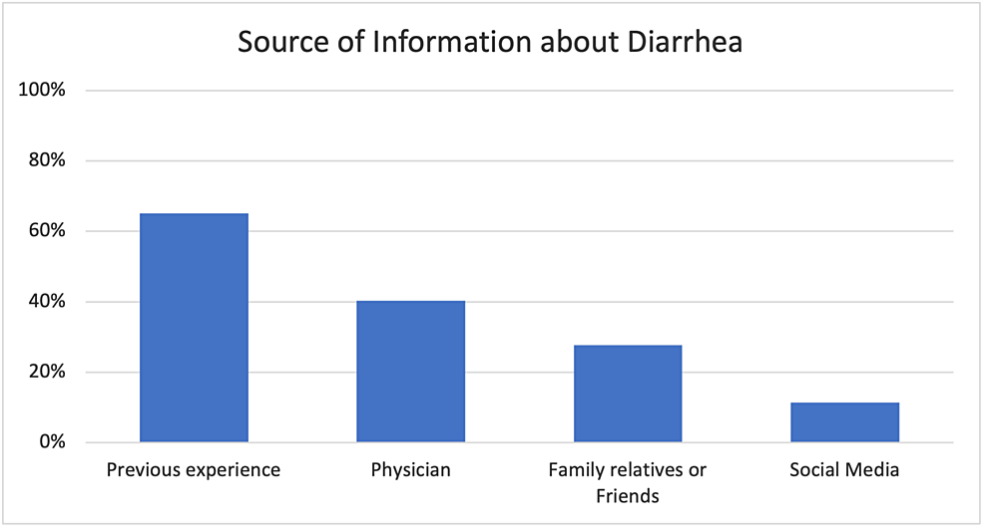
Source of information about diarrhea

**Figure 2:**
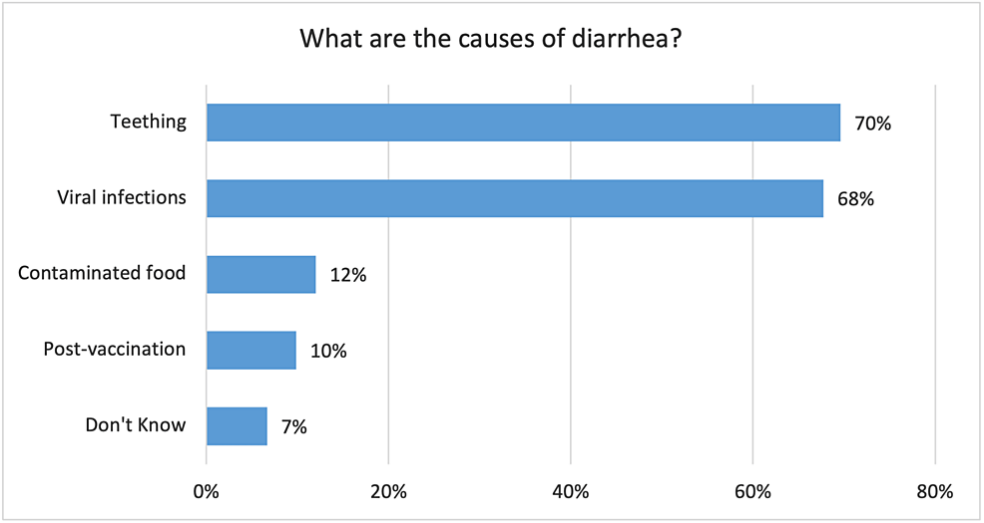
Mothers' perception of the common causes of diarrhea

Most of the participants defined diarrhea correctly (96.5%). Regarding the source of information about diarrhea, 65% of the mothers acquired their knowledge from their previous experience whereas 40% attained it from a physician. Figure 1 shows mothers’ source of information about diarrhea. When the mothers were asked about the possible causes of diarrhea, 261 (69.6%) of them believed that teething was the main cause of diarrhea followed by viral infections (67.7%), contaminated food (12%), and post-vaccinations (9.9%) (Figure 2).

**Figure 3:**
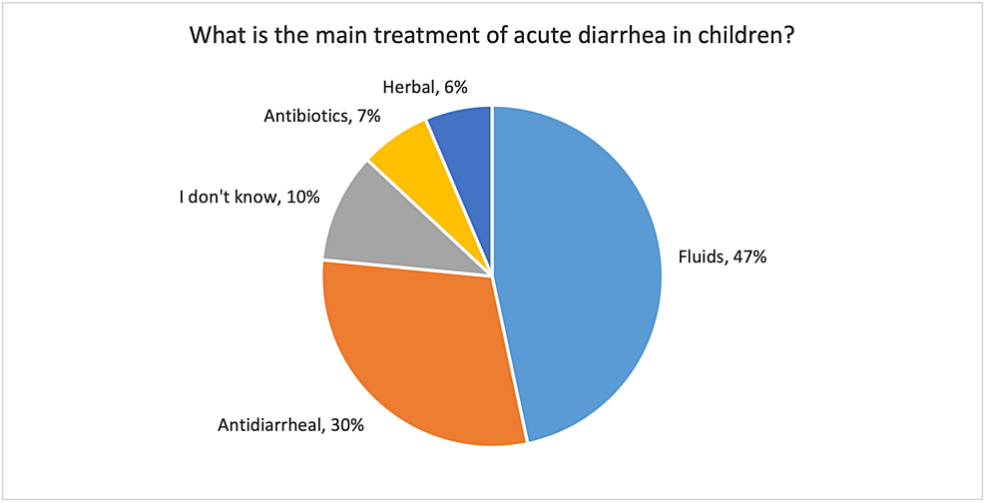
Mothers' perception of the treatment of acute diarrhea

A total of 175 mothers (47%) recognized fluid as the main treatment of diarrhea in children, however, 29.9% of them believed that antidiarrheal agents were the main treatment. Figure 3 demonstrates the main treatment of diarrhea as chosen by the participants. Regarding ORS, 92% were aware of it. In terms of the management of dehydration that is associated with diarrhea, 42.7% of the respondents thought that intravenous (IV) fluids were superior to ORS while 37% believed ORS to be more effective than IV fluids, and the rest (20%) assumed that they were equal. Only a few numbers (9.6%) of caregivers knew about the presence of vaccines which can reduce the incidence of diarrheal illnesses in children.

Practices

**Figure 4:**
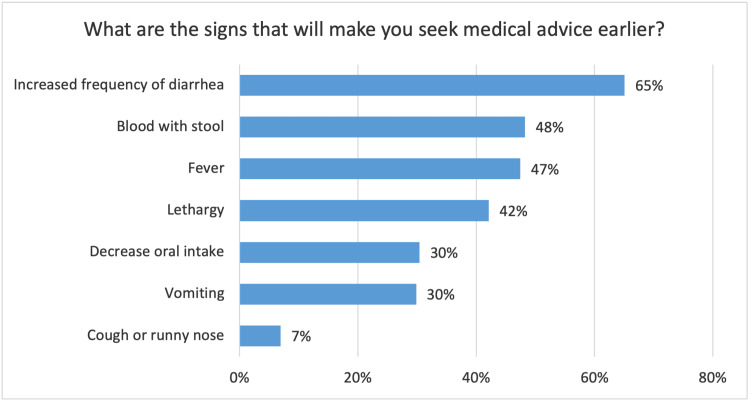
Mothers' reasons to seek medical care for children with acute diarrhea

Most of the mothers (84.3%) would give their kids more fluids during the diarrheal episodes, however, only 5.6% of the mothers would give fewer fluids. ORS was the main type of fluid (74.1%) chosen by the mothers to be given during the diarrheal episode, followed by plain water (62.7%), milk (12.8%), and herbal remedies (8.5%). Figure 4 shows that the increased frequency of diarrhea was the main reason that would make the mothers visit the hospital if the child has diarrhea, followed by blood in the stool (48.3%), lethargy (42.1%), and decreased oral intake (30.4%).

## Discussion

This study focused on exploring mothers’ knowledge and practices of a very common illness that is known to be a major cause of emergency department visits and hospitalization. The results showed that the majority of the participants had excellent knowledge of the definition of diarrhea, however, the majority cited their previous experiences as the primary source of information on diarrhea. This illustrates an area of improvement for physicians to educate caregivers about acute diarrhea and how to appropriately manage it at home.

When asked about the causes of acute diarrhea, around 70% of the mothers chose teething as the main cause followed by viral infections. Similar answers were found in multiple studies published in the literature which were conducted in multiple parts of the world [14-16]. This is a common misconception in the community which should be addressed and corrected since there is no supporting scientific evidence for this belief.

The main treatment of diarrhea was thought to be fluids by around 47% of mothers, nevertheless, 30% believed antidiarrheal agents to be the main treatment of acute diarrhea in children. In fact, the use of antidiarrheal agents in the setting of acute diarrheal illness can harm children and might result in multiple serious complications [17]. The use of antidiarrheal medications by the mother for treatment of acute diarrheal illnesses in children was reported by multiple studies which evaluated home-related management for diarrheal illnesses by the mothers [18,19].

The majority of respondents in our study were aware of ORS, which is a similar finding to the results of other studies [20,21]. Near half of the mothers interviewed in our study assumed that IV fluids were more effective than ORS in treating dehydration associated with diarrheal illnesses. This finding is similar to a study done in Eastern Ethiopia [13]. Such belief may lead to unnecessary visits to emergency departments in hope of receiving IV fluids even though mild diarrheal illnesses can be managed at home with oral fluids. This practice can overwhelm emergency departments and increase the burden on the health care system [22,23].

The introduction of the rotavirus vaccine decreased the incidence of rotavirus-related gastroenteritis among children significantly [24,25]. Unfortunately, most of the participants lacked knowledge about the presence of a rotavirus vaccine that can reduce the incidence of acute diarrheal illnesses in children. We need to educate caregivers more about such vaccines and their role in preventing common diarrheal illnesses in children.

In the second part of the survey, which focused on practices during diarrheal episodes, most of the mothers in our study would give more fluids than usual to their kids during diarrhea episodes and ORS was the main type of fluid. Compared to the Eastern Ethiopian study, they observed that mothers reduce the amount of fluids during diarrhea [13]. This could be related to the level of education since most of our included mothers were university graduates. Also, they might have easy access to information through Internet, books, and social media.

What is concerning is that even with a high level of education there were still around 8.5% of mothers who were using herbal remedies for managing diarrheal illnesses and that could result in dangerous consequences due to multiple factors such as types of herbs, lack of evidence, unclear mixing and preparation, purity of the herbs, and the effects on multiple body organs including liver and kidneys [26,27].

Limitations

However, the present study has limitations that may have affected our conclusions. This was a cross-sectional study conducted in one hospital and the results cannot be generalized to the whole country. Another drawback is the inherent defect in the cross-sectional study design, which does not account for changes in respondents' knowledge and attitudes over time, nor for the response and recollection bias inherent to this study design. Nonetheless, it is essential to highlight that the questionnaire was created and distributed in Arabic and that the translation may have contained inaccuracies. Lastly, most of the study participants were highly educated which may not represent the true population.

## Conclusions

Although the mothers interviewed showed an acceptable level of knowledge and practices regarding acute diarrheal illness in children, our study showed multiple issues. Specifically, the belief that teething was a cause of diarrheal illnesses, the lack of knowledge regarding rotavirus vaccines, and the idea that IV fluids were superior to ORS in the treatment of diarrhea-related dehydration.

To reduce diarrhea-related morbidity and mortality in children, specific attention must be given to improve maternal knowledge and practices regarding acute diarrheal illness. Measures like health awareness campaigns in the community, utilizing social media, and education by healthcare workers during hospital or clinic visits will make an impact on children's health.

## Appendices

Original questionnaire

Part 1 - Demographic characteristics

1) Nationality:

Saudi

Non-Saudi 

2) Age in years:

18-29

30-39

40 and above

3) Educational status:

Illiterate 

Primary School

Intermediate School

High School

University College 

Postgraduate studies

4) Number of children

1-2 children

3-4 children

5+ children

Part 2 - Knowledge

Q1

What is diarrhea? 

A. Frequent passing of watery stool (three or more times)

B. Frequent passing of well-formed stool

C. Blood in stools

D. Greenish stools

E. No idea

Q2

From where did you obtain your information about diarrhea? (You can choose multiple answers)

A. Physicians

B. Social media

C. Family relatives or friends

D. Previous experience

Q3

What are the possible causes of diarrhea?

(You can choose multiple answers)

A. Teething

B. Post-vaccination

C. Viral infections

D. Contaminated food

E. I do not know

Q4

What is the main treatment of acute diarrhea in children? (You can choose multiple answers)

A. Antibiotics

B. Antidiarrheal

C. Fluid

D. Herbal medication

E. I do not know

Q5

Have you ever heard about oral rehydration solution (ORS)?

A. YES (1)

B. NO (0)

Q6

There is a Vaccine included in the regular vaccination schedule that can reduce the occurrence of diarrhea in children?

A. YES

B. NO

Q7

Regarding the effectiveness of intravenous fluid and oral rehydration solution in the treatment of moderate dehydration associated with diarrhea?

A. IVF is better

B. ORS is better

C. Both are equal

Part 3 - Practice

If your child has diarrhea:

Q1

How frequently do you give fluid?

A. More than usual amount

B. The same amount

C. Less than usual amount

Q2

Which type of fluid do you give? (You can choose multiple answers)

A. Milk

B. Water

C. ORS

D. Herbal

Q3

What are the signs which will make you seek medical care earlier?

(You can choose multiple answers)

A. Lethargy

B. Increased frequency of diarrhea

C. Bloody diarrhea

D. Vomiting

E. Cough or runny nose

F. Fever

G. Decrease oral intake
